# Errata

**Published:** 1882-08

**Authors:** 


### ERRATA.

Dr. Rushmore desires us to state that the Amm. carb.$^{cm}$, given in case reported by him last March (p. 145), should have been marked (F. C.), not (F.).

Dr. Fincke, in the notation of his potencies, uses m for thousand and M for million. Therefore, on page 301, for Lachesis 2 m (F.), read Lachesis 2 M.

